# The new trochanteric nail TFN-A has a higher complication rate in the treatment of proximal femoral fractures than the predecessor model PFN-A

**DOI:** 10.1007/s00068-026-03304-3

**Published:** 2026-10-15

**Authors:** Hannes Kuttner, Michel Schläppi, Christoph Meier, Emanuel Benninger, Peter Wahl

**Affiliations:** 1 Division of Orthopaedics and Traumatology, Cantonal Hospital Winterthur, Brauerstrasse 15, Winterthur, 8401 Switzerland; 2 Department of Orthopaedic Surgery and Traumatology, Inselspital, University Hospital of Bern, Freiburgstrasse 18, Bern, 3010 Switzerland; 3 Present Address: Division for Orthopaedics and Traumatology, Cantonal Hospital Graubünden, Loestrasse 170, Chur, 7000 Switzerland; 4 ARTORG Center for Biomedical Engineering Research, Faculty of Medicine, University of Bern, Murtenstrasse 50, Bern, 3008 Switzerland; 5 Department of Biomedical Engineering, University of Basel, Hegenheimermattweg 167b, Allschwil, 4123 Switzerland

**Keywords:** Trochanteric fracture, Hip fracture, Proximal femur, Geriatric trauma, Cephalomedullary nail, Complications

## Abstract

**Aims:**

Trochanteric fractures (TF) are among the most frequent fractures and have major impact on the affected patients and the healthcare system. Cephalomedullary nails (CMN) are widely used for these fractures. Complications remain frequent, either due to comorbidities or concerning the implant. The goal of this retrospective, single-centre study was to compare the complication rates of two CMN from one manufacturer.

**Materials and methods:**

All internal fixations TF performed between 01/2017 and 07/2021 in our institution were analysed retrospectively. Follow-up was at least two years. The CMN were the PFN-A or the TFN-A. Demographic data, all reoperations and revisions were recorded. Reoperations and revisions were estimated using the Kaplan-Meier method, with subgrouping as minor reoperation, minor revision or major revision, including conversion to total hip arthroplasty.

**Results:**

A total of 492 CMN, used in 475 patients, could be included. Among these, 248 were PFN-A and 244 were TFN-A. The overall reoperation rate was 15%, 12.9% for the PFN-A and 17.2% for the TFN-A, respectively. The TFN-A group had significantly more major revisions (hazard ratio 3.57 (95% confidence interval 1.42–9.94; *p* = 0.004). No relevant differences were found regarding demographic data or the quality of reduction and internal fixation.

**Discussion:**

The TFN-A had a significantly higher rate of complications leading to major revision than the former PFN-A. This may have been missed when considering only the reoperation rate for all causes due to a camouflage effect by minor complications.

## Introduction

Trochanteric fractures (TF) are among the most frequently occurring fractures. Especially in the elderly, they have devastating effects on the patients’ quality of life and represent a major socioeconomic burden [1, 2]. Furthermore, these fractures are of special importance in developed countries due to demographic changes with ageing populations. In Germany, the yearly incidence in 2019 was as high as 108.7 per 100’000 inhabitants and showed an increase of 24% over the past decade [3]. Worldwide, a total of 1.66 million hip fractures, which encompass both TF and femoral neck fractures, were estimated to have occurred in 1990, and it was estimated that this number would rise to about 6.26 million in 2050 [4]. TF are not only very common but have complication rates after surgical treatment of up to 28.5%, or even of up to 50% when medical complications like pneumonia are taken into consideration [5–11].

Different strategies are available to treat these fractures. Cephalomedullary nails (CMN) are commonly used for this indication, as they have beneficial biomechanical properties, particularly advantageous in unstable fractures compared to sliding hip screw (SHS) systems [12–14]. Many different CMN from various manufacturers are available on the market, varying in technical details such as availability of screws or blades for fixation of the femoral head-neck fragment, the caput-collum-diaphyseal (CCD) angle, the mechanism allowing dynamic sliding of the screw, the number of screws, or regarding the curvature of the nail. The TFN-A (Trochanteric Femoral Nail Advanced, DePuy Synthes, Oberdorf, Switzerland) is an evolution of the TFN (Trochanteric Fixation Nail, DePuy Synthes), in turn an evolution of the PFN-A (Proximal Femoral Nail Antirotation, DePuy Synthes) and of the original PFN (Proximal Femoral Nail, DePuy Synthes). According to the manufacturer, benefits of the new system would be a bone-preserving shape of the proximal part of the nail, a smaller radius of curvature to reduce impingement of the tip at the anterior cortex, improved static locking options and enhanced cement augmentation possibilities [15].

So far, there is no evidence of significant differences between the various types of trochanteric CMN regarding clinical outcomes. The TFN-A has been used since May 2019 in our institution, replacing the PFN-A, which had previously been used. This retrospective, single-centre study compared the outcomes of both CMN regarding reoperation rates and mortality. The null hypothesis was that there was no difference in outcomes between both CMN.

## Materials and methods

### Study design

This was a single-centre, retrospective, clinical, observational study. No investigation was performed for study purposes. As the patients or their legal representatives had provided written informed consent for data analysis and as the analysis was performed on anonymized data, the study fulfilled the national legal requirements for a quality control study, waiving the requirement for submission to the regional ethical committee.

### Data collection

Cases treated for femoral fractures were identified by checking the in-house electronic patient billing database for ICD-10 codes containing S72. XX between 1st January 2017 and 31st July 2021. All cases were screened manually to identify the use of a CMN, excluding other treatment modalities as well as pathological fractures.

Data were collected from the electronic patient records established during follow-up examinations or from other departments within the same institution. Demographic data like sex, age, body mass index (BMI) and American Society of Anesthesiology (ASA) classification were also collected from the patient files. Patients who had no recorded follow-up at two years postoperatively were contacted by phone, if they were unable to attend the routine follow-up examination. If necessary, relatives or the treating general practitioner (GP) were contacted to identify reoperations performed elsewhere.

Outcome parameters were all types of reoperations within 24 months and patient survival. Reoperations without exchange of any components and diaphyseal dynamization were considered as minor reoperations. Reoperations with exchange of the cervical blade/screw only, or definitive removal of the CMN were considered as minor revisions. Reoperations with exchange of the entire CMN, conversion to total hip arthroplasty (THA) and internal fixations due to peri-implant fractures were considered as major revisions. Conversion to THA was also considered separately. Indications for reoperation/revision/conversion were noted. Any date of death was also cross-checked with the federal death registry [16].

The fractures were classified following the AO-/ASIF classification, without using subgroups [17]. Bone quality was assessed using the Singh index [18–20]. Implant length, CCD angle, type of cervical fixation device (screw or blade), length of screw/blade and use of cement augmentation were noted. The quality of reduction and fixation were classified using the Baumgartner criteria [21], the Cleveland zones for screw position [22] and the tip-apex-distance (TAD) [23]. All measurements and assessments were performed on pre- and intraoperative radiographs by the first author (H.K.).

### Statistical analysis

All statistical analyses were performed using R version 4.4.2 (R Foundation for Statistical Computing, Vienna, Austria). The cases were stratified according to the CMN used, either the PFN-A or the TFN-A.

For univariate comparisons between the PFN-A and the TFN-A groups, variables were summarized with median and range for continuous variables and counts and percentages for categorical variables. Differences between groups were assessed using the Wilcoxon rank-sum test for continuous variables and the Chi-squared test or Fisher’s exact test for categorical variables, as appropriate. To account for multiple testing in the univariate comparisons, p-values were adjusted using the Bonferroni correction.

Survival analysis was performed using the Kaplan-Meier method to estimate 24-month revision rates. Cox proportional hazards models were used to compare the two groups and calculate hazard ratios (HRs) with 95% confidence intervals (CI) for the following events: any reoperation, minor reoperation, minor revision, and major revision. Statistical significance was defined as a p-value < 0.05.

## Results

In total, 2’586 cases with femoral fractures were identified within the study period between 1st January 2017 and 31st July 2021. Of these, 492 CMN were used in 475 patients. The other patients had been treated using other implants or had to be excluded due to the presence of a pathological fracture. Among these CMN the PFN-A was used in 248 cases and the TFN-A in 244 cases.

**Fig. 1:**
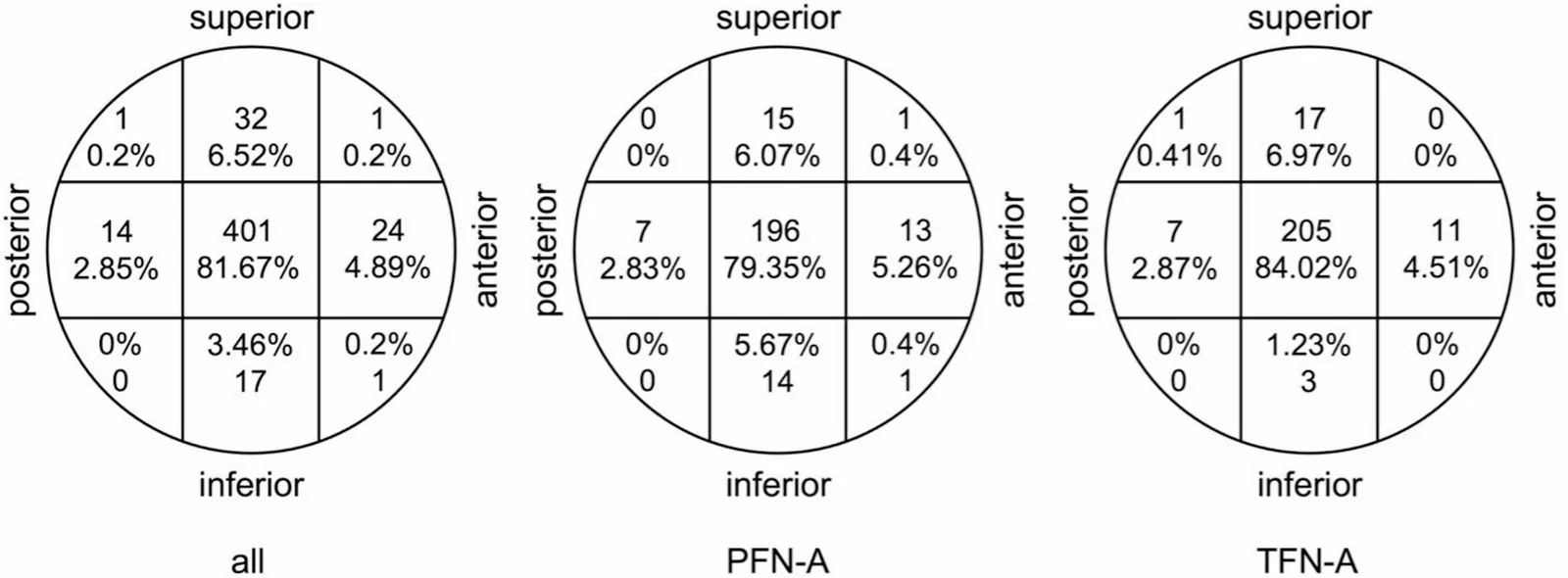
Detailed distribution of the screw/blade position according to the Cleveland zones for the whole study group (all) and separated for the subgroups PFN-A and TFN-A

Demographic and surgical data of the study cohort are summarized in Table 1, the detailed distribution of the Cleveland zones is visualized in Fig. 1. Demographic data showed no relevant differences between the groups. BMI was missing in 8 cases. No follow-up data beyond hospitalisation could be obtained for 4 patients, as neither the patient nor the relatives or the GP could be reached.

**Table 1:** Demographic data, nail specifications, classifications and quality parameters of the whole study group (all), and the subgroups (PFN-A and TFN-A)

	all	PFN-A	TFN-A	*p*-value
**Demographics**				
sex, n (%)				0.112
female	351 (71.3%)	185 (74.6%)	166 (68.0%)	
male	141 (28.7%)	63 (25.4%)	78 (32.0%)	
Age, years median (min; max)	85 (32; 107)	85 (32; 107)	85 (39; 102)	0.919
BMI, kg/m^2^ median (min; max)	23 (13;41)	23 (13; 38)	23 (13; 41)	0.317
ASA, n (%)				0.734
I	1 (0.2%)	1 (0.4%)	0 (0.0%)	
II	109 (22.2%)	54 (21.8%)	55 (22.5%)	
III	312 (63.4%)	159 (64.1%)	153 (62.7%)	
IV	69 (14.0%)	34 (13.7%)	35 (14.3%)	
V	1 (0.2%)	0 (0.0%)	1 (0.4%)	
Singh-classification, n (%)				0.835
I	35 (7.1%)	19 (7.7%)	16(6.6%)	
II	154 (31.3%)	79 (31.9%)	75 (30.7%)	
III	247 (50.2%)	119 (48.0%)	128 (52.5%)	
IV	48 (9.8%)	26 (10.5%)	22 (9.0%)	
V	8 (1.6%)	5 (2.0%)	3 (1.2%)	
VI	0 (0.0%)	0 (0.0%)	0 (0.0%)	
**Nail specifications**				
nail length, n %)				0.678
short (170 & 200 mm)	324 (65.9%)	166 (66.9%)	158 (64.8%)	
long (≥ 260 mm)	168 (34.1%)	82 (33.1%)	86 (35.2%)	
nail diameter, n (%)				0.595
9 mm	3 (0.6%)	2 (0.8%)	1 (0.4%)	
10 mm	69 (14.0%)	31 (12.5%)	38 (15.6%)	
11 mm	89 (18.1%)	50 (20.2%)	39 (16.0%)	
12 mm	261 (53.0%)	132 (53.2%)	129 (52.9%)	
14 mm	69 (14.0%)	32 (12.9%)	37 (15.2%)	
CCD, n (%)				< 0.001
125°	203 (41.3%)	177 (71.4%)	26 (10.7%)	
130°	289 (58.7%)	71 (28.6%)	218 (89.3%)	
cervical fixation method, n (%)				0.245
Screw	2 (0.4%)	0 (0.0%)	2 (0.8%)	
Blade	490 (99.6%)	248 (100.0%)	242 (99.2%)	
screw/blade length, mm median (min; max)	95 (75; 125)	95 (75; 110)	95 (75; 125)	0.098
cement augmentation, n (%)	15 (3.0%)	0 (0.0%)	15 (6.1%)	< 0.001
**Classification and quality parameters**				
AO/ASIF classification, n (%)				0.171
31-A1	128 (26.0%)	62 (25.0%)	66 (27.0%)	
31-A2	240 (48.8%)	126 (50.8%)	114 (46.7%)	
31-A3	88 (17.9%)	41 (16.5%)	47 (19.3%)	
diaphyseal (32xx)	31 (6.3%)	14 (5.6%)	17 (7.0%)	
combinations	5 (1.0%)	5 (2.0%)	0 (0.0%)	
Baumgartner criteria, n (%)				0.616
Good	201 (40.9%)	101 (40.7%)	100 (41.0%)	
Acceptable	289 (58.7%)	145 (58.5%)	144 (59.0%)	
Poor	2 (0.4%)	2 (0.8%)	0 (0.0%)	
TAD, mm median (min; max)	19 (5; 37)	19 (6; 34)	19 (5; 37)	0.327
Cleveland zones, n (%)				0.092
superior posterior	1 (0.2%)	0 (0.0%)	1 (0.4%)	
superior center	32 (6.5%)	15 (6.0%)	17 (7.0%)	
superior anterior	1 (0.2%)	1 (0.4%)	0 (0.0%)	
center posterior	14 (2.8%)	7 (2.8%)	7 (2.9%)	
center center	401 (81.5%)	196 (79.0%)	205 (84.0%)	
center anterior	25 (5.1%)	14 (5.6%)	11 (4.5%)	
inferior posterior	0 (0.0%)	0 (0.0%)	0 (0.0%)	
inferior center	17 3.5%	14 5.6%	3 1.2%	
inferior anterior	1 (0.2%)	1 (0.4%)	0 (0.0%)	

Concerning fracture classification, quality of reduction and fixation as well as nail specifications, no significant differences were found, except in two categories. First, a statistically significant difference was found for the CCD (*p* < 0.001), as 125° was used 177 (71.4%) times in the PFN-A group and 26 times (10.7%) in the TFN-A group, while 130° was used 71 (28.6%) times and 218 (89.3%), respectively. Secondly, cement augmentation was never used in the PFN-A group and 15 times (6.1%) in the TFN-A group (*p* < 0.001).

**Fig. 2:**
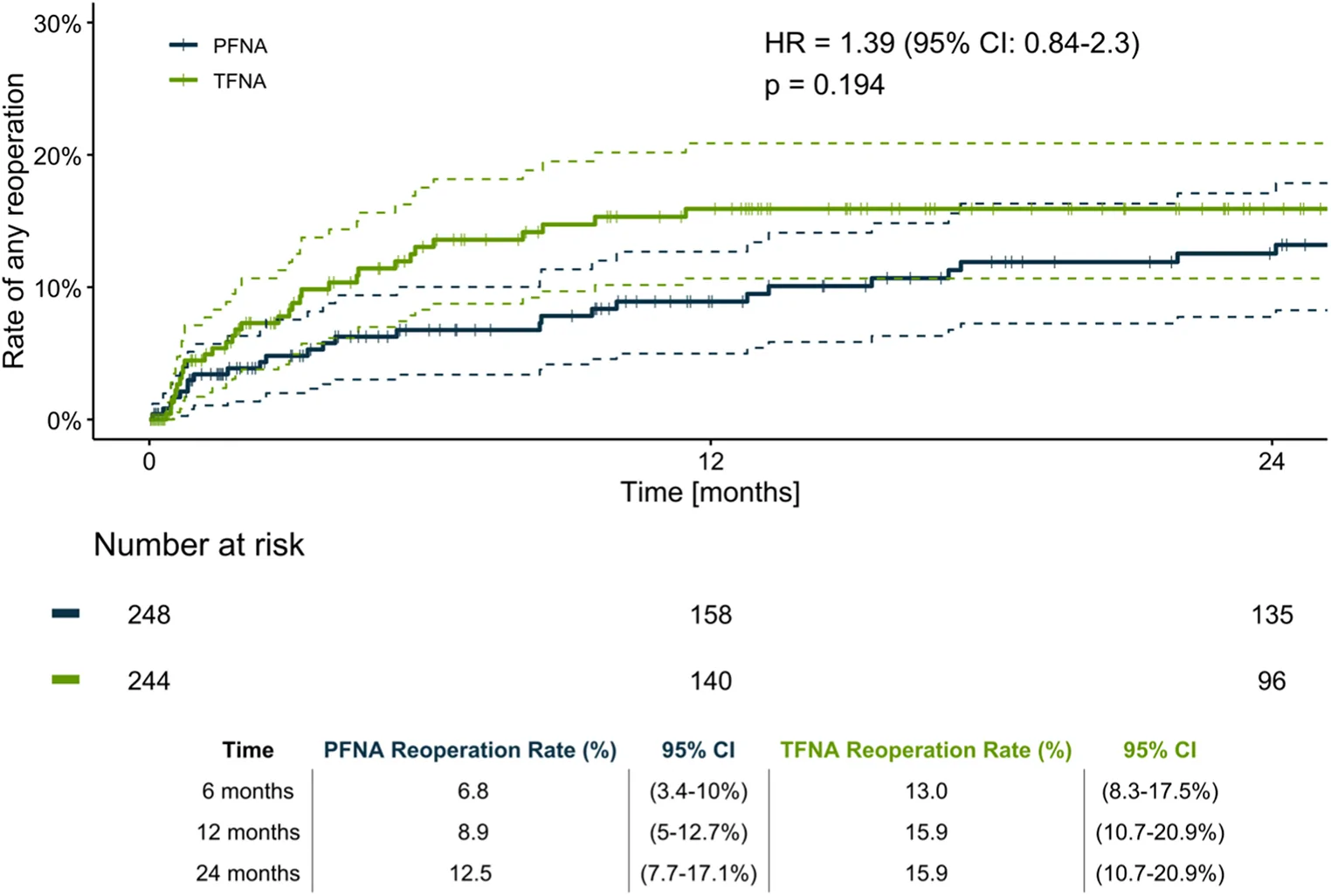
Kaplan-Meier curve for the event “any reoperation”

**Fig. 3:**
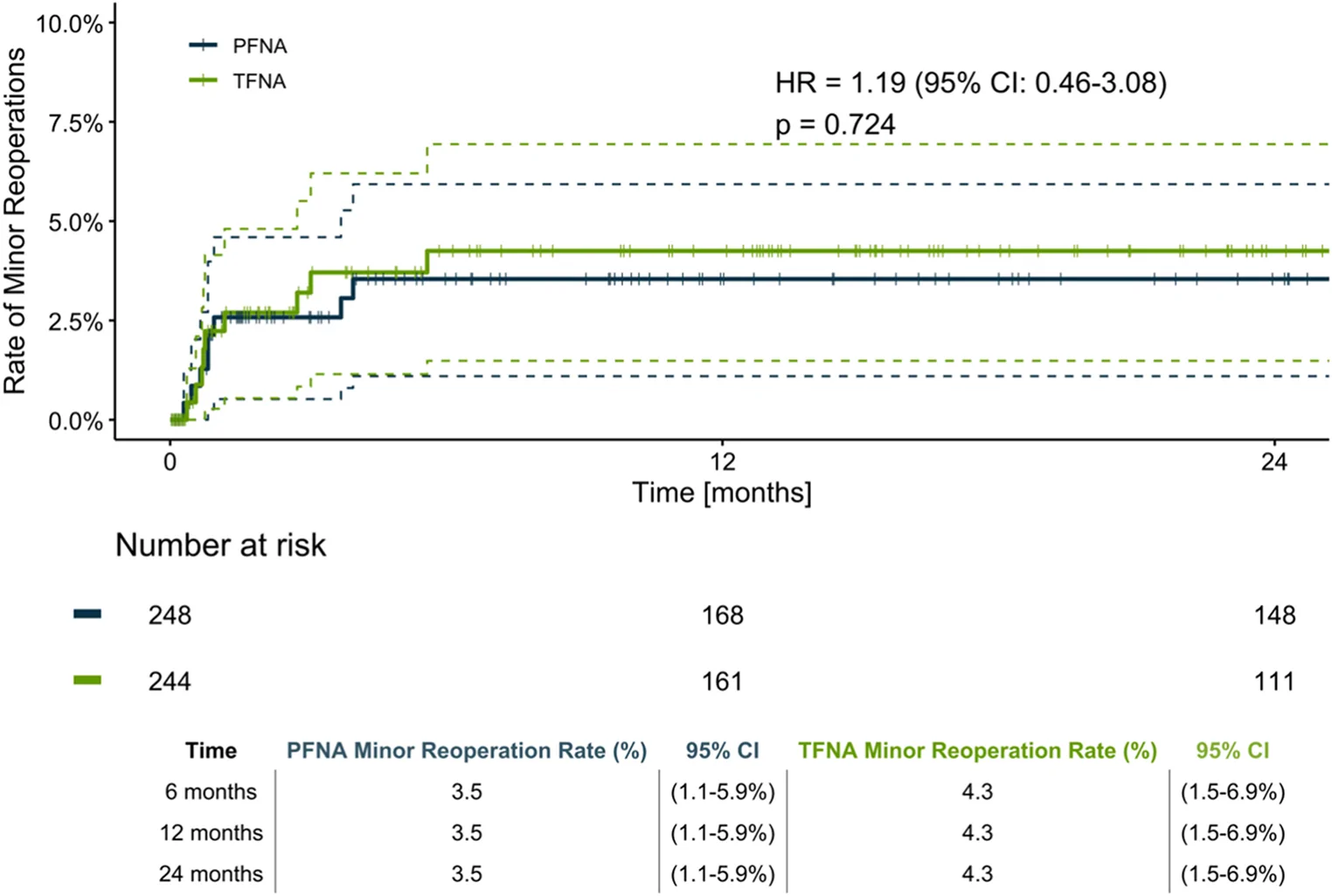
Kaplan-Meier curve for the event “minor reoperation”

**Fig. 4:**
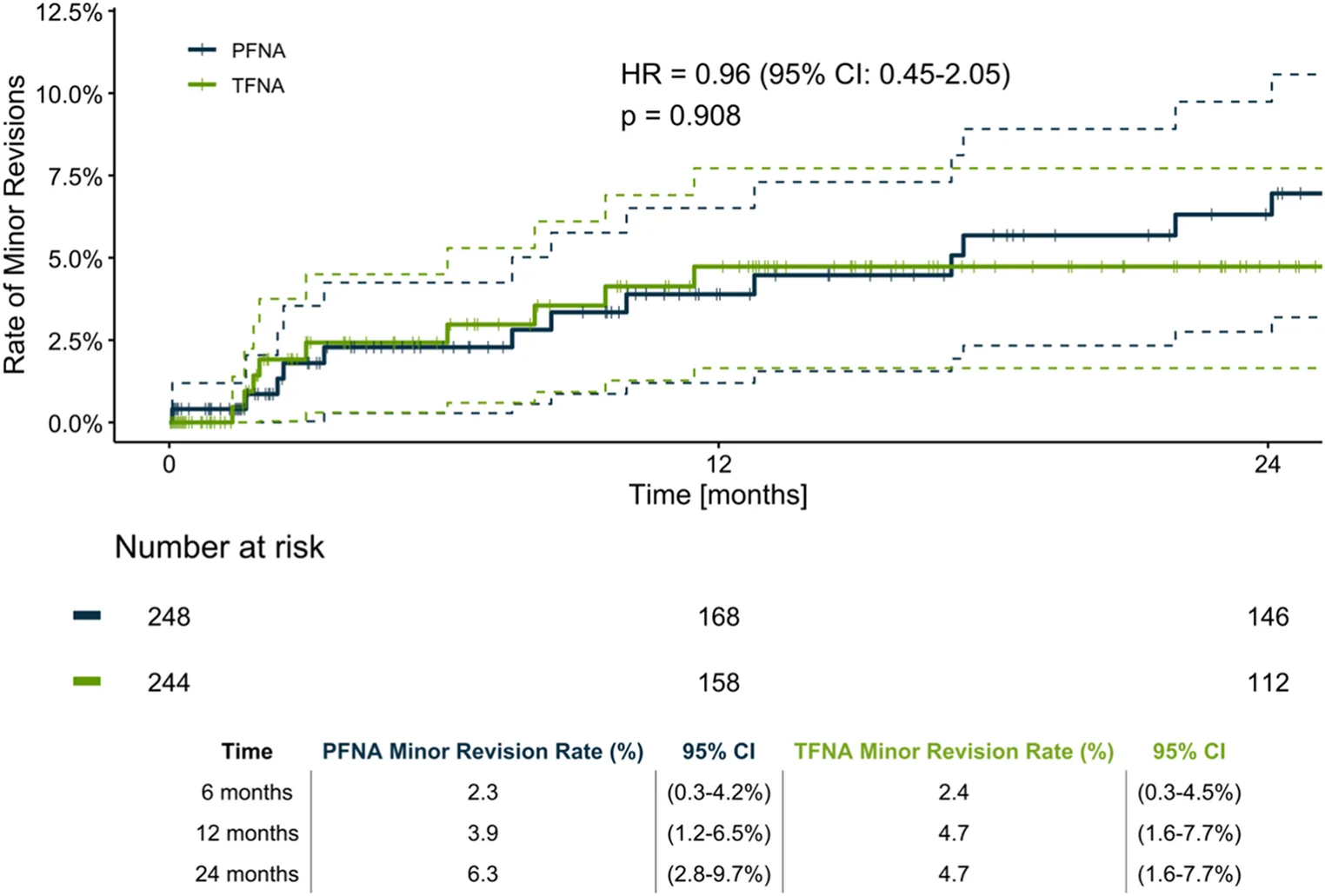
Kaplan-Meier curve for the event “minor revision”

**Fig. 5:**
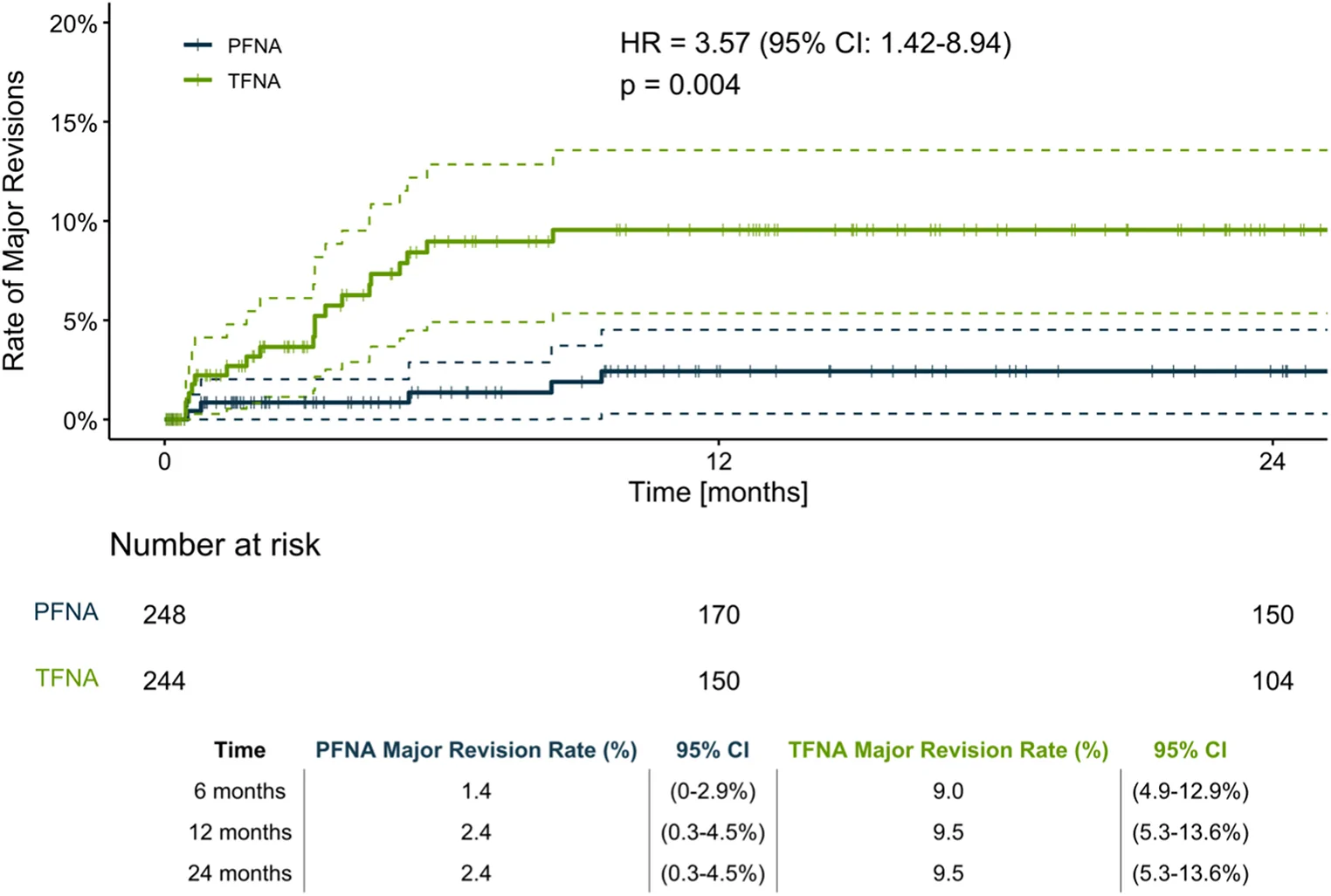
Kaplan-Meier curve for the event “major revision”

Details of the registered reoperations, the distribution among the subcategories, minor reoperation, minor revision and major revision, as well as reasons for reoperation are provided in Table 2. No statistically significant differences were found regarding the total number of reoperations, minor reoperations and minor revisions (Figs. 2, 3 and 4). However, the TFN-A was associated with more major revisions compared to the PFN-A (*p* = 0.004) (Fig. 5). Among these, conversion to THA was performed 6 times in the PFN-A and 11 times in the TFN-A group. Nail breakage was observed less frequently with the PFN-A (*n* = 3; 1.2%) than with the TFN-A (*n* = 7; 2.9%) (*p* = 0.143). The median (range) time to reoperation was 1 (0 to 24) month for the entire cohort, respectively 2 (0 to 24) months in the PFN-A group and 1 (0 to 11) month in the TFN-A group (*p* = 0.136). Of the 475 patients treated, 30.1% (*n* = 143) died within the first year.

**Table 2:** Detailed distribution of reoperations and diagnoses leading to reoperation at 24 months follow-up within the whole study group (all) and the subgroups (PFN-A and TFN-A)

	all	PFN-A	TFN-A	*p*-value
**Detailed distribution of reoperations, n (%)**				
any reoperation	74 (15.0%)	32 (12.9%)	42 (17.2%)	0.194
minor reoperation	23 (4.7%)	11 (4.4%)	12 (4.9%)	0.724
minor revision	24 (4.9%)	13 (5.2%)	11 (4.5%)	0.908
major revision	27 (5.5%)	8 (3.2%)	19 (7.8%)	0.004
**Detailed distribution of diagnoses leading to reoperation**,** n (%)**				
cut-through/cut-out	18 (3.7%)	5 (2.0%)	13 (5.3%)	0.086
wound problems	6 (1.2%)	4 (1.6%)	2 (0.8%)	0.686
delayed union/non-union	7 (1.4%)	4 (1.6%)	3 (1.2%)	1.000
nail breakage	10 (2.0%)	3 (1.2%)	7 (2.9%)	0.143
fracture-related infection	14 (2.8%)	5 (2.0%)	9 (3.7%)	0.399
lateral pain	8 (1.6%)	5 (2.0%)	3 (1.2%)	0.724
symptomatic osteoarthritis	3 (0.6%)	2 (0.8%)	1 (0.4%)	1.000
peri-implant fracture	1 (0.2%)	0 (0.0%)	1 (0.4%)	0.496
limb length discrepancy	1 (0.2%)	0 (0.0%)	1 (0.4%)	0.496
patients’ request	5 (1.0%)	3 (1.2%)	2 (0.8%)	1.000
other	1 (0.2%)	1 (0.4%)	0 (0.0%)	1.000

## Discussion

TF are among the most frequently occurring fractures, especially among geriatric patients [1, 2]. Different treatment modalities are available, with CMN being one of the most frequently used methods worldwide. As the TFN-A was introduced in May 2019 in our institution, the aim of this retrospective analysis was to compare the complication rates of the new implant with those of the previously used PFN-A. During the study period, between January 2017 and July 2021, 492 CMN were used in 475 patients. Among these, 248 were PFN-A and 244 were TFN-A. To our knowledge, the current study includes the largest sample size comparing TFN-A to PFN-A. Although we could not observe any statistically significant difference regarding the total number of reoperations for both implants, there was a significantly higher number of major revisions in the TFN-A group, mostly due to cut-through/cut-out or more frequent implant breakage. Complications leading to reoperations occurred mostly during the early postoperative period.

The quality of reduction and of the internal fixation was not significantly different between both groups, when considering the Baumgaertner criteria, the Cleveland zones and the TAD [21–23]. This is essential to allow a comparison of outcomes linked to the implant. The quality of the reduction was rated as “acceptable” only in 58.7% of the cases, which seems a relatively high proportion, whereas “good” was achieved in 40.9% only. A reason for this lies in the definition of the Baumgaertner criteria, which considers two parameters, the alignment and a displacement of more than 4 mm of any fragment. If one of these criteria is not met, it results in the category “acceptable”. As avulsions of the lesser trochanter are not addressed routinely, reduction many AO/ASIF type 31-A2 fractures, the most frequent type affecting about half the patients (Table 1), can only be rated as acceptable, even though the fragments relevant for the stability were reduced properly [17, 21]. When ignoring pure avulsions of the lesser trochanter (not fractures of the posteromedial facet) quality of reduction had been rated as “good” in 55.5% of the cases and “acceptable” in 44.1%, respectively.

Concerning specificities of the implants, two differences were present. The standard CCD used in the PFN-A group was 125°, whereas it was 130° in the TFN-A group. A higher CCD may lead to difficulties in achieving a correct placement of the cervical screw with a regular TAD, in particular if some residual varus displacement is present or if there is a marked varus of the femoral neck, which occurs frequently in osteoporotic patients [24, 25]. Nevertheless, if this would have been a relevant problem in our study population, it should have resulted in a difference in TAD or Cleveland zones. While a finite element analysis suggests a disadvantageous load transmission on the fracture when using a higher CCD, other biomechanical studies found that load distribution on the implant is favourable when loaded in valgus rather than varus [26, 27]. Furthermore, reduction in neutral position or even slight valgus is the key to correct treatment of trochanteric fractures, as it provides more favourable force transmission and as it allows compensation for shortening due to impaction of the fracture zone [27–30]. Despite the lower CCD, which may be less advantageous regarding load transmission on the implant or locking of the sliding mechanism, less frequent nail breakage was observed with the PFN-A (*n* = 3; 1.2%) than with the TFN-A (*n* = 7; 2.9%). This may also not be explained by the material, as the PFN-A implants were made of stainless steel, whereas the TFN-A were made of titanium, a material with a higher fatigue resistance [31]. Therefore, the higher implant fracture rate observed with the TFN-A indicates a less favourable design of the implant.

Cement augmentation is known to improve pull-out resistance and rotational stability of cervical screws and blades [32–34]. In the study cohort, it was used in only 15 TFN-A cases and in none of the cases treated with a PFN-A. Among the augmented cases, no reoperations were detected. Therefore, this difference had no negative influence on the study outcomes.

In total, we detected 74 reoperations, accounting for 15.0% of all cases. We detected no difference between the groups when considering all types of reoperations, with *n* = 32 (12.9%) vs. *n* = 42 (17.2%) for the PFN-A and the TFN-A, respectively. This lies within the range of rates reported in the literature, even though comparison is difficult, as different definitions of complications have been used in existing reports [5–11, 35, 36]. Despite the retrospective nature of the study, it may be expected that very few reoperations were missed, if any, as elderly patients in Switzerland usually seek revision care at the nearest hospital [37].

We defined three types of reoperations, using recognized standards in arthroplasty regarding this terminology: minor reoperation, minor revision, and major revision, the latter including conversion to THA, nail exchange and internal fixation due to peri-implant fractures. While we found no significant differences between the PFN-A- and the TFN-A groups regarding any reoperations, minor reoperations and minor revisions, a significant difference was seen for the major revisions. This underscores the importance of proper differentiation of the severity of the complication, to avoid any camouflage effect due to minor events. While 8 (3.2%) major revisions occurred in the PFN-A group, there were 19 cases (7.8%) in the TFN-A group (*p* = 0.004). When focussing on the reasons for the higher rate of major revision, more frequent implant breakage and more frequent cut-through/cut-out were present (Table 2). With the adaptations of the TFN-A, higher fatigue strength in biomechanical testing compared to previous models were shown. One might expect, that this would result in lower rates of TFN-A breakage in the clinical setting. However, this could not be confirmed in our study [15]. All fractures occurred at the aperture for the blade/screw, matching the observations of previously published analyses of TFN-A breakage [38]. One possible explanation might be a weakening of the implant at the aperture for the blade or screw induced by drilling/reaming over the cervical guide wire, which is often slightly deflected by the lateral cortex during insertion. Furthermore, the reduced proximal diameter may lead to inferior stabilisation of the fracture, which can cause higher loads on the proximal part of the nail. As the smaller proximal nail diameter may also cause a greater amount of toggling of the implant within the medullary cavity of the femur, this might explain a higher rate of medial migration of the blade [39]. Furthermore, the blade and the screw of the TFN-A also have a smaller diameter compared to the PFN-A, potentially reducing the resistance under varus stress and medial migration [40]. All these factors together may explain the higher rate of cut-out/cut-through observed in this cohort for the TFN-A. We did not investigate the effect of the learning curve associated with the introduction of a new implant, as another study showed no or only minimal influence on the performance of the TFN-A [41]. Since we did not observe any differences regarding quality of reduction and implant placement, we do not think that the learning curve has a decisive influence.

As limitations of our study, the retrospective design and the low statistical power have to be mentioned. Strengths of our investigation are the equally distributed study groups and that all cases were treated within the same setting. Furthermore, information regarding reoperations, even through relatives or the GP, could not be obtained for only four patients, representing less than 1%.

## Conclusion

This study shows the importance of systematic outcome measures to evaluate the performance of different implants. The adaptations made in the newer CMN TFN-A might lead to a higher rate of major revision than the older design PFN-A. This should be taken into consideration, especially when using the nail in unstable TF. Establishing national or international fracture registries or databases would allow gathering the much-needed data for more precise analysis.

## Data Availability

All data supporting the findings of this study are available within the paper and its supplementary information.
